# Variation in Social Feeding Behaviors and Interactions Among *Caenorhabditis Nematodes*

**DOI:** 10.1002/ece3.72522

**Published:** 2025-11-16

**Authors:** Dustin Haskell, Jhelaine Palo, Reina F. H. Eugene, Christopher R. L. Large, Michael P. Hart

**Affiliations:** ^1^ Department of Genetics, Perelman School of Medicine University of Pennsylvania Philadelphia Pennsylvania USA

**Keywords:** behavior, Caenorhabditis, evolution, interactions, nematode, social

## Abstract

The ability to respond to stimuli and environmental cues is essential for higher order organisms to survive and reproduce and requires a neuronal network that can integrate cues and execute behavioral responses. Evolution of behaviors occurs ubiquitously in most established ecological niches, even among closely related species. To uncover the genetic and neuronal drivers of evolving behaviors, we have taken advantage of the large and relatively ancient divergence in the *Caenorhabditis* genus to ask how different *Caenorhabditis* nematodes respond to environmental stimuli and whether behavioral traits are shared or distinct. Here, we assayed foraging behaviors of 12 members of the *Caenorhabditis* clade, including members of both the *elegans* and *japonica* a subgroups, and the basal taxon *C. monodelphis*. For each species, we analyzed social feeding and food bordering behaviors, which are well characterized in *C. elegans* . These behaviors are the functional readout of complex sensory integration of multiple sensory cues including pheromones, touch, O_2_/CO_2_ concentration, and attractive and noxious stimuli. We hypothesized that the evolutionary divergence between species would correlate with divergence in these behaviors. We observed a wide variation in social aggregate feeding and bordering behaviors of hermaphrodite and female animals, but the variation did not correlate with the evolutionary relatedness of the species. The addition of male animals with female or hermaphrodite animals of the same species increased the aggregation behavior of a subset of species, but not other species. The combination of a second species with *C. elegans* significantly reduced the aggregate feeding behavior of *C. elegans* , but not the other species, suggesting intraspecies and interspecies interactions modify behaviors. Overall, we find that foraging and social feeding behaviors vary widely across *Caenorhabditis* species, likely due to species‐specific responses and integration of environmental and contextual sensory cues. The *Caenorhabditis* clade represents a compelling model to dissect the evolution of behavior across diverse environments and a large timescale.

## Graphical abstract

Different species of roundworms (nematodes) are frequently found together in the wild and can aggregate in groups while feeding. We find that different species of worms display a range of feeding behaviors, from social to solitary, unrelated to evolutionary relatedness. Population context can change social feeding behaviors within species, where mixing sexes can increase, and mixing species can decrease, social feeding. Therefore, environmental and population variables strongly influence social behaviors.

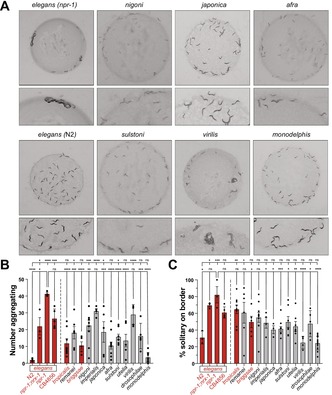

## 1 Introduction

Behavior is how organisms interact with their environment and respond to both internal and external stimuli. Simple forms of life, especially those that represent unicellular phyla, rely heavily on the detection of chemical and tactile stimuli through direct contact (Bagorda and Parent 2008). In contrast, sensory specialization in Metazoans through the evolution of differentiated and highly specialized neuronal tissues, results in a complex breadth of behavioral responses to the environment (Archibald 2015; Valencia‐Montoya et al. 2024). The evolution of complex behavior can be loosely correlated with the evolution of multicellularity and complex tissues (Wan and Jékely 2021). Tissue differentiation gave rise to neuronal cells and tissues, which link networks of sensory cells together to form a basic neuronal circuit. In more complex Eukaryotes, sophisticated neuronal networks utilize multiple sensory and neuronal cell types to form the basis of complex neuronal ganglion, nerve tracts, and the brain (Baluška and Mancuso 2009; Hartenstein and Stollewerk 2015).

Changes in behavioral traits occur in most eukaryotic organisms, especially between related species (Martins 1996). Rapid shifts in behavior are tightly coupled with the alteration of an organism's physiologic and genetic drivers, highlighted by several studies in *Drosophila* and related species (Ding et al. 2019; Hernández et al. 2021). Small changes in gene regulatory networks, modest alterations in neuronal circuits, or mutations influencing receptor/ligand interactions, are all sufficient to alter behaviors in an often unpredictable manner. Without the ability to identify stepwise drivers of shifting behaviors, it can be challenging to understand how selective pressures lead to specific modifications of genetic and molecular factors, which dictate the neuronal and circuit activity that underlies complex responses to environmental stimuli.

Compounding the study of behavioral evolution is the complex nature of behavior itself. In line with Tinbergen's fundamental questions, to truly differentiate between behavioral paradigms requires leveraging both physiological and evolutionary dissection at multiple levels of organismal complexity, starting with changes to the genome and epigenome, and culminating in intra‐circuit alterations that modify the complex network of integrated and often codependent behaviors (Bergman and Beehner 2022). While comprehensive dissection of genetic and molecular drivers of altered or novel behaviors is rarely achieved, we can utilize existing genetic information in clades such as *Caenorhabditis* to begin circuit‐level descriptions of behavior at this level.

**FIGURE 1:**
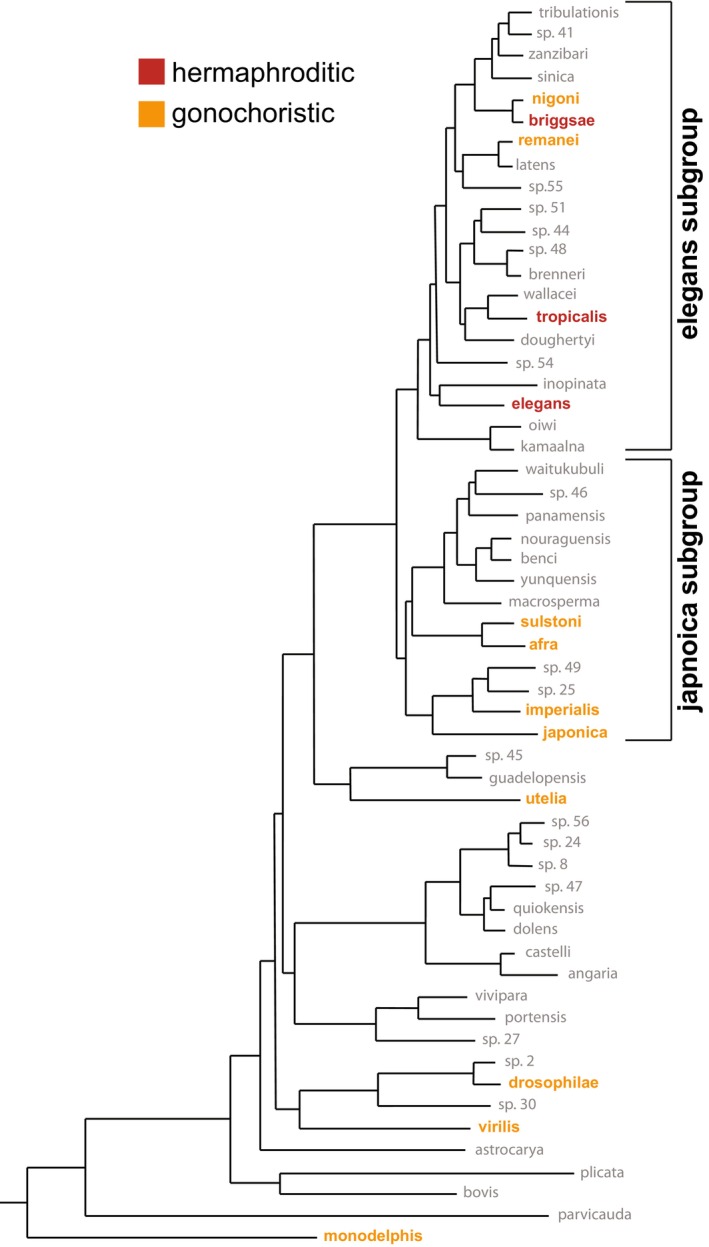
The *Caenorhabditis* clade of nematodes is an evolutionary and diverse lineage. (A) Phylogenetic tree of *Caenorhabditis* clade showing the two large subgroups (*elegans* and *japonica*) and the basal taxon *C. monodelphis*. Species included in behavioral assays are highlighted and color‐coded based on reproductive strategy (red = hermaphroditic or orange = gonochoristic). Figure was adapted from Stevens et al. (2020).

Among common lab organisms, the nematode *Caenorhabditis elegans* ( *C. elegans* ) is a particularly powerful tool for studying neuronal circuits and behaviors. In addition to many available genetic tools, a fully mapped neuronal connectome (including chemical, electrical, and peptide signaling) (Bhattacharya et al. 2019; Ripoll‐Sánchez et al. 2023; Cook et al. 2019), allows stereotyped behaviors to be directly linked to the activity of specific neurons and circuits. In addition, the *Caenorhabditis* clade of nematodes provides a unique opportunity to study the evolution of behavior in a diverse and evolutionarily ancient lineage (Figure 1) (Stevens et al. 2020). Nematodes of this clade are found in a widespread terrestrial distribution, with populations found in nearly every ecological niche probed for their presence (Cutter 2015). In the wild, members of the *Caenorhabditis* nematodes persist in a free‐living state colonizing and utilizing bacteria‐rich substrates as a food source, such as rotting fruit. The global dispersion of individual members of the clade means they experience significant variation in the local environment, including differences in temperature, humidity, concentration of oxygen, and microbiota (Schulenburg and Félix 2017). Notably, *Caenorhabditis* species are rarely isolated from homogenous populations; for example, *C. elegans* is frequently co‐isolated with *C. briggsae* and *C. remanei* from the same substrate. Altogether, the *Caenorhabditis* clade presents a model to query the evolution of behavior across a large evolutionary timescale and a wide range of environments.

Due to high levels of variation among local environments, we chose to assay foraging behaviors, which we reasoned would be present across the clade due to similar or shared feeding strategies. One well‐characterized *C. elegans* feeding behavior is social feeding, and it is defined by social interactions that occur between individual animals feeding in the same local environment (de Bono and Bargmann 1998). One can speculate that this behavior has costs and benefits for individuals and populations of animals, although this is not well defined. Animals may be attracted to high‐density food that is resource‐rich for nutrient seeking, but animals populating a food/bacteria‐rich location can also spread and increase the food source (bacteria) to surrounding areas. Further, group feeding could facilitate short‐distance signaling between animals (perhaps through pheromones) and/or increase opportunities for mating, but this behavior is also environment and context dependent (de Bono et al. 2002; Moreno et al. 2016). Wild *C. elegans* isolates show a surprisingly high level of variability in social feeding behaviors. Contrary to the widely used N2 Bristol lab strain, which displays solitary feeding, wild isolates from a range of environments show higher levels of social feeding (de Bono and Bargmann 1998). de Bono and Bargmann defined the solitary feeding behavior of the N2 Bristol strain to a missense mutation in the *npr‐1* gene (ortholog of neuropeptide Y receptor) resulting in an amino acid change in the third intracellular loop of the NPR‐1 G‐protein‐coupled receptor (de Bono and Bargmann 1998). Loss of function mutations in the *npr‐1* gene result in high levels of social feeding in *C. elegans* (de Bono and Bargmann 1998). Here, we utilize the *npr‐1(ad609)* loss of function mutation to induce social feeding in the N2 Bristol lab control strain as a reliable and robust positive control for social feeding behaviors observed in wild isolate *C. elegans* strains (Cowen et al. 2024).

Subsequent work elucidated the role of NPR‐1 and other neuronal drivers of solitary and social feeding, including that *C. elegans* social feeding behavior results from the integration of environmental cues including pheromones, O_2_/CO_2_ concentrations, and perception of touch and noxious stimuli (de Bono et al. 2002; Macosko et al. 2009; Laurent et al. 2015; Cheung et al. 2005; Bretscher et al. 2008). Sensory neurons including ADL, ASH, ASK, ADE, and AWB form a circuit to detect the local sensory environment, which is then integrated through the interneuron RMG to alter locomotion paradigms (Macosko et al. 2009; Laurent et al. 2015). Genetic dissection of this sensory circuit has uncovered distinct roles for the neuropeptide receptor NPR‐1 (de Bono and Bargmann 1998; Cowen et al. 2024) and gap junction connections between sensory neurons and the RMG interneuron (Macosko et al. 2009; Jang et al. 2017). More recent work has implicated glutamate signaling from ADL and ASH sensory neurons, where conserved synaptic adhesion molecules (NRX‐1 and NLG‐1) contribute to social feeding, and *nrx‐1* impacts the architecture and function of ASH chemical synaptic connections (Cowen et al. 2024). Therefore, social feeding behavior relies on codependent signaling of neuropeptides, and electrical and chemical synapses, which together fine‐tune the balance between solitary and social feeding (Cowen et al. 2024). Given the complexity of this behavioral circuit, we asked whether social feeding behaviors are conserved throughout the *Caenorhabditis* clade of nematodes.

In this study, we compared social feeding behaviors of 12 *Caenorhabditis* species to multiple strains of *C. elegans* (Figure 1). This clade of nematodes is not only relatively ancient, but also has significant divergence over evolutionary time, with an estimated genomic divergence of > 20 million years between *C. elegans* and the four most closely related species (Memar et al. 2019). Two large subgroups, *C. elegans* and *C. japonica*, have emerged based on genomic data (Stevens et al. 2020). *C. monodelphis*, represents the basal taxon of the clade and is estimated to have diverged over 100 million years ago, an evolutionary timescale far beyond that between mice and humans (Ponting and Goodstadt 2009). Lastly, a major strength of this model lies in the opportunity to study “wild” populations of nematodes (representing both hermaphroditic and gonochoristic species) in a controlled lab setting applicable to medium‐throughput behavioral assays. We therefore set out to identify differences among the species that represent evolution of behavior within the *Caenorhabditis* clade, and that will provide a foundation for future investigation into the underlying differences in sensory cues, neuronal function, and circuitry.

We found significant variation in social feeding behaviors of hermaphrodites and females across the 12 members of the *Caenorhabditis* clade, with all but two showing reduced levels of aggregate feeding compared to the *C. elegans* social feeding control strain, and half showing increased social feeding compared to the *C. elegans* solitary control strain. In testing more ecologically relevant populations in our assays, we also identified the impact of intraspecies and interspecies interactions. Mixing males with females (or hermaphrodites) of individual species altered aggregate feeding behavior in two species when compared to homogeneous female (or hermaphrodite) populations. Combining a second species with social feeding *C. elegans* sufficiently significantly reduced aggregate feeding of *C. elegans* . This represents the first characterization of foraging and social feeding behaviors across the *Caenorhabditis* clade of nematodes and lays the groundwork for the dissection of ecological, cellular and circuit‐level mechanisms of conserved behaviors.

## 2 Methods

### 2.1 Strains Maintenance

Animals were maintained under normal growth conditions (~20°C) on normal NGM media (Brenner 1974). Plates were seeded with *E. coli* OP50 to provide food. Strains utilized in this study are listed in Table 1. *npr‐1*(*ad609*) and *nrx‐1*(*wy778*) mutations were confirmed in *C. elegans* strains and were used as positive controls. All other species were obtained from the CGC and maintained by chunking.

**TABLE 1:** Reference table for *Caenorhabditis* species information.

Species	Subgroup	Strain	RS	GH	Genome accession	FMC	Behavioral characterization
*elegans*	*elegans*		⚥	Lab strain	PRJNA13758	Yes	Yes
N2							
CB4856							
*briggsae*	*elegans*	AF16	⚥	Lab strain	PRJNA10731	Yes	Yes
*imperialis*	*elegans*	EG5942	MF	30× inbred	GCA_963572205.1	n/a	No
*nigoni*	*elegans*	JU1422	MF	25× inbred	PRJNA384657	n/a	No
*remanei*	*elegans*	PB4641	MF	20× inbred	PRJNA577507	Yes	Some
*tropicalis*	*elegans*	JU1373	⚥	n/a	PRJNA53597	n/a	No
*japonica*	*japonica*	DF5081	MF	20× inbred	GCA_963572235.1	Yes	No
*afra*	*japonica*	JU1286	MF	20× inbred	GCA_963570955.1	n/a	No
*sulstoni*	*japonica*	JU2788	MF	25× inbred	PRJEB12601	n/a	No
*virilis*	*drosophilae*	JJU1968	MF	25× inbred	GCA_964036255.1	n/a	No
*drosophilae*	*drosophilae*	DF5112	MF	20× inbred	PRJEB67483	n/a	No
*uteleia*	*guadalopensis*	JU2585	MF	25× inbred	GCA_963573275.1	n/a	No
*monodelphis*	n/a	JU1667	MF	20× inbred	GCA_964197825.1	Yes	No

*Note:* ⚥ represents hermaphroditic species.

Abbreviations: GH, genetic heterogeneity number of times inbred according to CGC strain information; MC, fine morphological characterization (characterization of fine morphological features and/or internal anatomy); MF, represents gonochoristic species; RS, reproductive strategy.

### 2.2 Social Feeding Behavior Assay

Standard 6‐well culture plates were filled with 6 mLs of standard NGM media and then seeded with 75 μL of *E. coli* OP50 and allowed to dry overnight. For the standard assay, 50 animals were picked onto clean plates and then moved onto the seeded wells, transferring as little bacteria as possible to prevent buildup. For standard social feeding and bordering assays, 50 animals were either female or hermaphrodites depending on the species. For the mixed sex assays, we combined 25 males with 25 females/hermaphrodites (depending on species). Regardless of the composition of the assay, worms were picked during and placed on the assay during their fourth larval stage. Given the subtle variations in developmental timing between species we selected animals for the assay on morphology alone. Female or hermaphrodite animals were staged based on the presence of the half‐moon shape characteristic of an L4 developing vulva. Males were staged based on relative size, germline features, and morphological development of the tail. After placing the animals on the 6‐well plate, a small amount of Tween20 was used to thinly coat the lid to prevent condensation forming during imaging. The plate is then imaged using the WormWatcher automated imaging platform (Tau Scientific) for a total of 20 h with a cluster of 10 images (over a 1‐min timecourse) being taken every hour. To quantify social feeding behavior each well was manually scored from blinded images, where a worm was considered aggregating if it was contacting two or more other worms (de Bono and Bargmann 1998; Cowen et al. 2024). Bordering was assayed by calculating the percentage of solitary animals in direct contact with the border region of the bacterial lawn (with the border defined as approximately half a body length in width). Data shown are from the 15‐h mark, which placed the animals of all species in the middle of Day 1 of adulthood.

### 2.3 Mixed Species Assays

In order to assay the effect of mixed species, we first generated a fluorescently tagged *C. elegans* strain to differentiate them from the other species while imaging behavior. *npr‐1(ad609) C. elegans* were injected using standard techniques with plasmid *myo‐2p*::*mScarlet* in order to label pharyngeal muscle (25 ng/μL). Strong expression of the transgene allows visualization of a single worm when in an aggregate. To perform the assay, a standard 5 cm NGM plate was seeded with 25 μL of *E. coli* OP50 and allowed to dry. To mix the populations, 15 *C. elegans* expressing *myo‐2p*::*mScarlet* were placed on the bacterial lawn along with 15 of *C. monodelphis* or *C. nigoni*. Smaller populations (*n* = 30) were used to maximize the resolution of inter‐worm interactions in images, thus allowing for careful quantification of aggregates. Due to the lack of fluorescent imaging in the WormWatcher setup, we instead placed plates under a fluorescent dissecting scope and allowed them to sit for 45 min to mitigate any effects of plate movement. The plate was then imaged using white light (for a reference image) and green light (~546 nm, to capture the *mScarlet* fluorescence). Consistent with previous assays, animals touching two or more individuals were scored as aggregating. Worms were imaged using a Leica M165FC dissecting scope equipped with a Cool LED fluorescence source and FlexCam C3 camera.

### 2.4 Orthology Analysis

We used the genome sequence and gene annotations from Wormbase (Sternberg et al. 2024) and the *Caenorhabditis* Genome Project to find orthologous gene sets (orthogroups) in non‐*elegans* *Caenorhabditis* species. First, we extracted the longest isoform from every gene using AGAT (Dainat et al. 2024). Then, using the protein sequence, we constructed orthogroups using OrthoFinder (Emms and Kelly 2015, 2019) using the sequence aligner, Diamond, in ultrasensitive mode (Buchfink et al. 2015).

### 2.5 Statistics and Reproducibility

The number for each experiment was based on previous studies and effect size, with each experiment performed with at least three independent replicates and each trial performed with matched controls. All data were analyzed and plotted in GraphPad Prism 10 and statistical significance was determined using one‐way ANOVA with Tukey's post hoc test. For comparisons of two datasets, a two‐tailed unpaired t‐test was used to compare significance. Standard linear regression analysis was performed to generate the line of best fit; *R* ^2^ and *p*‐values are given. Error bars on figures represent the standard error of the mean (SEM) and *p*‐values are shown in each figure to indicate significance (*p* < 0.05).

## 3 Results

### 3.1 Social Feeding Behaviors Differ Across the *Caenorhabditis* Clade

**FIGURE 2:**
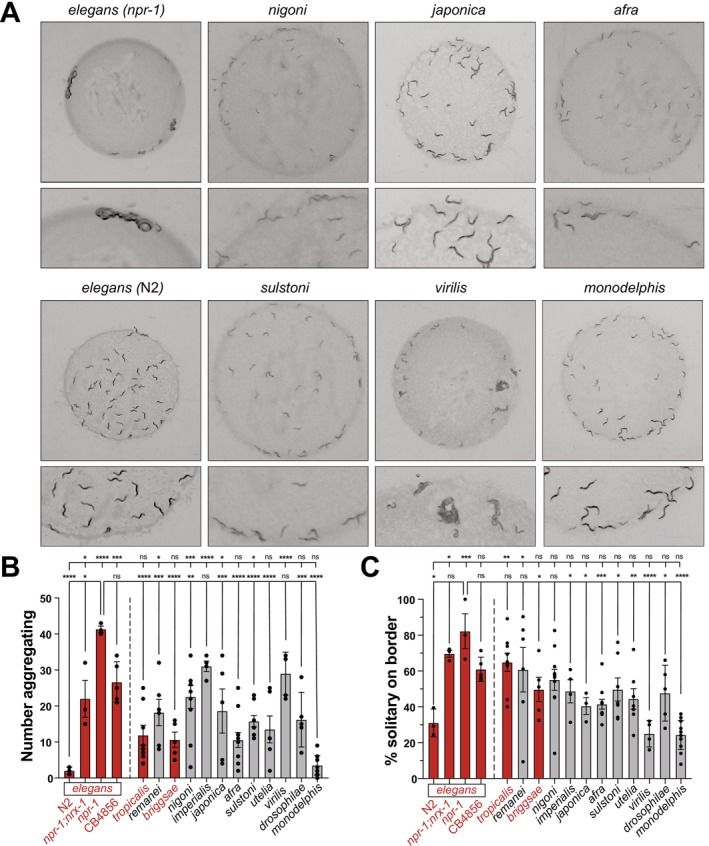
Aggregation behavior is diverse across *Caenorhabditis* clade. (A) Representative images of select *Caenorhabditis* species aggregation feeding and bordering behaviors. (B) Quantification of number of aggregating animals in 12 members of the *Caenorhabditis* clade. Species are ordered based on the provided phylogeny (and for subsequent figures). Significant variation is present among members of the clade, and also within individual species. The four *C. elegans* groups assayed represent the negative control (N2 “Bristol”), positive control *npr‐1(ad609)*, modulated positive control *npr‐1(ad609*);*nrx‐1*(*wy778*), and wild isolate CB4856 “Hawaiian,” respectively. Bar graphs represent the mean values across biological replicates (each replicate indicated by a dot). Error bars show stand error of mean. One‐way ANOVA with Tukey's post hoc test was used for statistical comparisons, with *p*‐values indicated in standard format (*p* = 0.05(*), *p* = 0.01(**), *p* = 0.001(***), and *p* = < 0.001 (****)). Statistical comparisons were made between *C. elegans npr‐1(ad609)* and all other strains (lower stats bar) and *C. elegans* N2 and all other strains (upper stats bar). Hermaphroditic species are labeled in red, while gonochoristic species are labeled in black. (C) Quantification of percent solitary animals on food border in 13 members of *Caenorhabditis* clade. Significant variation is present among members of the clade, and also within individual species. Graphical representations and statistical analysis are consistent with previous panel.

We assayed aggregation and bordering behavior using a quantitative imaging setup (Cowen et al. 2024). Assays were performed as previously described, with 50 female animals used in species that are gonochoristic. We included four *C. elegans* control strains: N2 “Bristol” which are solitary feeding; *npr‐1(ad609)* which are social feeding; and *npr‐1(ad609);nrx‐1(wy778)*, and CB4856 “Hawaiian” *C. elegans* which display intermediate social feeding. To observe trends due to species relatedness, the species were ordered based on their phylogenetic relationship and position. We observed wide variation in mean aggregation behavior among members of the genus, with some species like *C. imperialis* and *C. virilis* showing relatively high levels of aggregation, although not to the level of the *C. elegans* social control ( *C. elegans* *npr‐1(ad609)*) (Figure 2A,B). Interestingly, *C. monodelphis* displays similar aggregation to the *C. elegans* solitary controls (N2) with little to no aggregation (Figure 2A,B). Beyond these three examples, we find that the majority of the species have an intermediate level of aggregation. Further, we observe variation between replicates/experiments within some individual species, namely *C. japonica, C. nigoni, C. uteleia, and C. remanei* (Figure 2A,B). Somewhat surprisingly, there was no clear pattern linking evolutionary relatedness and the observed level of aggregation. In addition to aggregating during social feeding behavior, the *C. elegans* aggregates tend to be near the border of the bacterial lawn where the bacteria are the thickest (Figure 2A) (de Bono and Bargmann 1998). We observed a downward trend in bordering behavior the more distantly related the species are to *C. elegans* , and large variation within some species in bordering behavior (Figure 2A,C).

**FIGURE 3:**
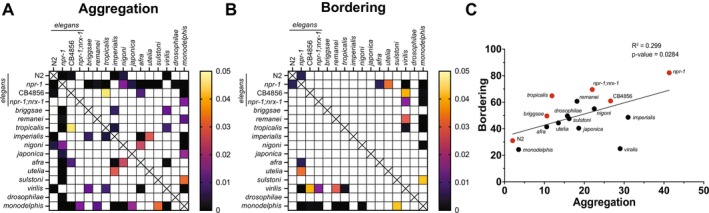
Statistical analysis of phenotypes. (A) Heat‐map of ANOVA‐generated *p*‐values of all pairwise comparisons in aggregation assay. Significance is indicated by color (see legend); white squares indicated any *p*‐value greater than a 0.05 threshold and therefore are not considered significant. (B) Heat‐map of ANOVA‐generated *p*‐values of all pairwise comparisons in bordering assay. (C) XY data plot showing clustering off species when comparing aggregation and bordering phenotypes. As anticipated, *C. elegans* N2 and *C. monodelphis*, and *C. elegans npr‐1* represent the low and high extremes of the cluster, respectively. Linear regression analysis shows a significant relationship. Consistent with the figures above, hermaphroditic species are labeled in red.

To visualize variation among species in both aggregation and bordering phenotypes, we generated heat‐maps of the adjusted *p*‐values generated by one‐way ANOVA of all combinatorial comparisons (Figure 3A,B, respectively). An XY plot reveals a broad dispersal without a clear clustering among closely related species (Figure 3C). Overall, the plot emphasizes that while *C. elegans* N2 and *npr‐1*, and *C. monodelphis* are relative extremes compared to the rest of the species, such extremes occur both in “domesticated” lab and “wild” populations. Consistent with their independent evolution of hermaphroditism (Kiontke et al. 2004), there does not seem to be any significant clustering of the hermaphroditic species. A linear regression analysis revealed a significant correlation between aggregation and bordering values (*p* = 0.0284), suggesting that these two behaviors are likely strongly linked.

### 3.2 Ecological Drivers Modulate Social Feeding Behaviors in a Species‐Specific Manner

**FIGURE 4:**
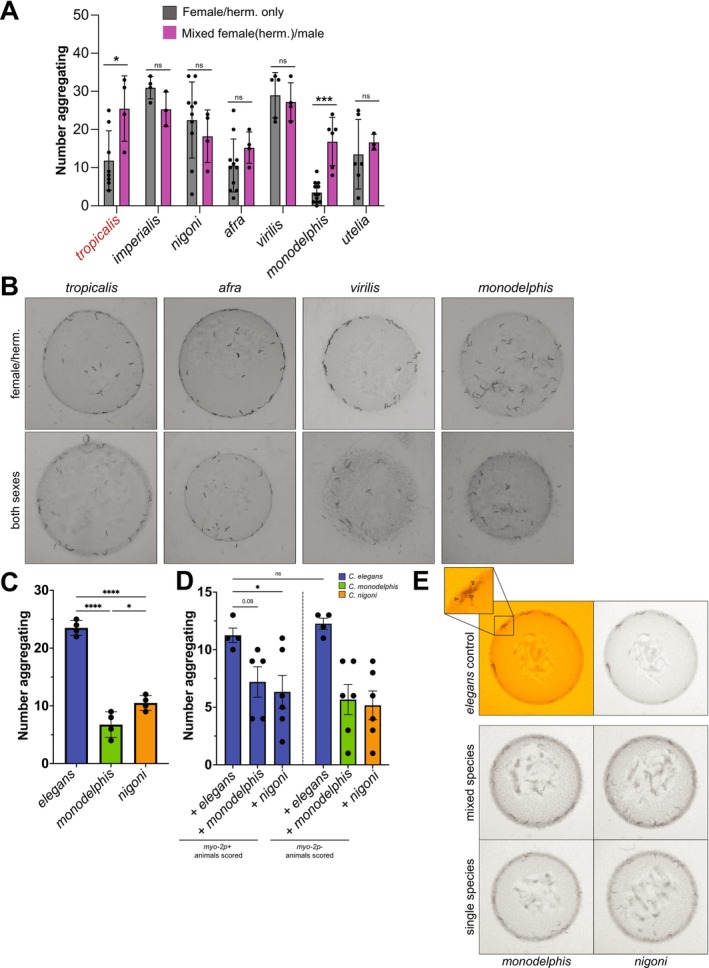
Ecological drivers modulate social feeding behaviors in a species‐specific manner. (A) Quantification of number of aggregating animals of *Caenorhabditis* species in mixed sex populations and representative images (B). *C. tropicalis* and *C. monodelphis* both showed significant differences in aggregation when males were added to the population. Bar graphs represent the mean values across biological replicates (each replicate indicated by a dot). Error bars show stand error of mean. Standard T‐test was used for individual comparisons; *p*‐values are indicated in standard format (*p* = 0.05(*), *p* = 0.01(**), *p* = 0.001(***), *p* = < 0.001 (****)). Hermaphroditic species are labeled in red, while gonochoristic species are labeled in black. (C) Quantification of 30 animal single‐species controls (D) Quantification of mixed species assays, where 15 *C. elegans npr‐1(ad609) myo‐2p +* animals were mixed with 15 *C. elegans npr‐1(ad609) myo‐2p‐, C. monodelphis*, or *C. nigoni*. All subsequent interspecies assays were done utilizing *C. elegans* *npr‐1(ad609)*. Combining *C. elegans* and *C*. *nigoni* significantly lowers aggregation of *myo‐2* expressing *C. elegans* compared to their *C. elegans* alone control (left), while addition of *C. monodelphis* caused an insignificant but trending reduction (left). *C*. *monodelphis* and *C*. *nigoni* have significantly lower aggregation than their *C. elegans* *myo‐2p*– control (right). (E) Representative images of mixed species assays. *myo‐2p* expressing *C. elegans* are indicated by arrows (top left window).

Given the differences in environment and reproductive strategies among the species, we reasoned that the makeup of the individual populations could influence social feeding behaviors. To test this hypothesis, we repeated the standard aggregation assays using mixed sex populations (25 males +25 females, or 25 hermaphrodites in hermaphroditic species with the presence of males) (Figure 4A,B). Overall, the ratio of males in the 10 male/female species is variable among the species tested and likely depends on a number of context‐dependent factors. For this assay, we selected seven species with a sufficient ratio of males present in the population, choosing representatives from across the full range of phylogeny, including a hermaphroditic species *C. tropicalis* and the basal taxon *C. monodelphis*. In addition, we selected a subset of the species displaying a high level of variation in the female/hermaphrodite only assays. We hypothesized that for a true male/female species (with a naturally higher number of males), a higher ratio of males within the assay may represent a more “ecologically relevant” population and therefore reduce some of the observed variation. Overall, for most species, the mixed sex populations did not alter aggregation behavior compared to the single sex populations (Figure 4A,B). However, *C. monodelphis* and *C. tropicalis*, showed significantly increased aggregation in the mixed sex population assay compared to the single sex hermaphrodite/female population assay (Figure 4A). These results again highlight the impact of local environment and context on variation in behaviors between species, whereas these assays reflect a modulated response to a real‐time sensory‐environment landscape.

To explore other ecologically relevant population dynamics (such as interspecies cohabitation and interactions, e.g., escape from predation (Quach and Chalasani 2020)), we combined additional species with *C. elegans* to test how this would influence *C. elegans* social feeding behavior. To test this, we used a reduced population size (30 animals) and the addition of a *myo‐2p* fluorescent transgene, which we found did not impact the aggregation behaviors of the species. Aggregation in both *C. nigoni* and *C. monodelphis* was significantly reduced compared to *C. elegans* (15 *myo‐2p* positive (+) and 15 *myo‐2p* negative (−)) (Figure 4C), and significantly different from one another, which is consistent with the 50 animal aggregation assay results (Figure 2). Furthermore, the inclusion of the *myo‐2p* transgene did not impact *C. elegans npr‐1*(*ad609*) aggregation behavior. Next, we mixed 15 *myo‐2p* + *C. elegans npr‐1(ad609)* social feeding controls with 15 *myo‐2p*‐ *C. elegans, C. monodelphis*, or *C. nigoni* on a seeded NGM plate and allowed the mixed populations to acclimate for 45 min. After the acclimation period, the plates were imaged and assayed for aggregation behavior. We aimed to understand if the mixing of two distinct populations would alter the aggregate feeding dynamics of either or both species. In the mixed species population assays, the addition of *C. nigoni* or *C*. *monodelphis* to the *myo‐2p* + *elegans* population significantly reduced the aggregation behavior of *C. elegans* (Figure 4D,E). Interestingly, we did not observe an impact of *C. elegans* on the aggregation behavior of the other species. Disruption of *C. elegans* aggregation behavior is likely the result of a number of changes to environmental cues present on the plates from mixing populations.

### 3.3 *Caenorhabditis* Clade Has High Level of Conservation in NPR‐1 Receptor Protein

**FIGURE 5:**
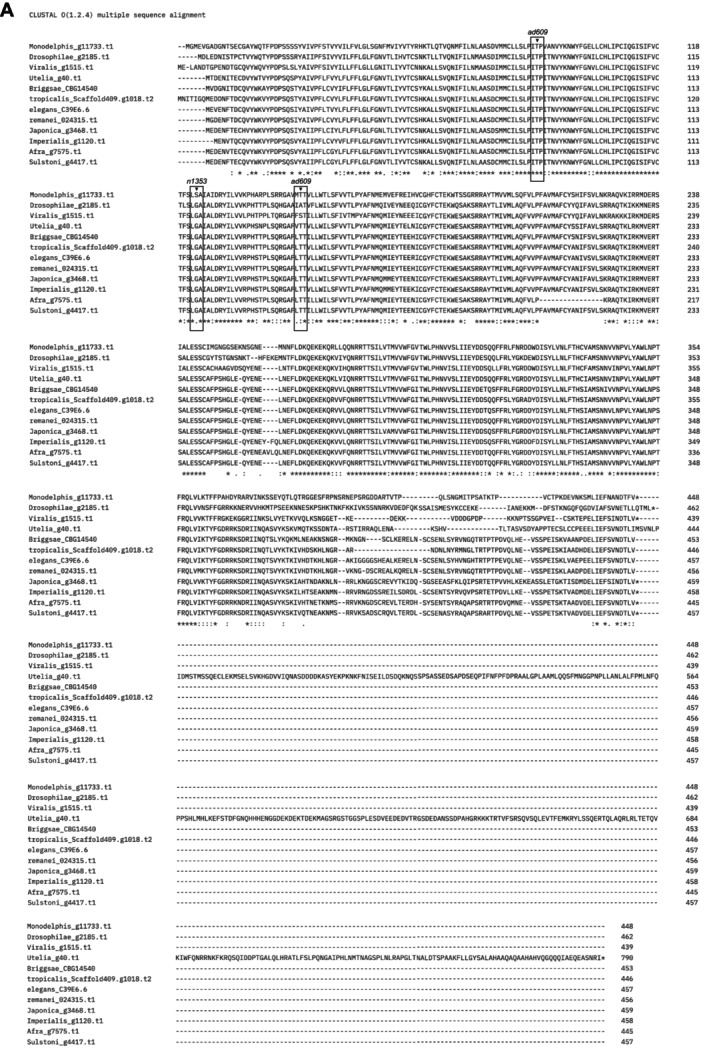
Protein alignment of NPR‐1 orthologs among *Caenorhabditis* nematode species. (A) NPR‐1 orthologs were queried from existing *Caenorhabditis* genomes or assemblies based on sequence similarity. A single ortholog was identified in each species with the exception of *C*. *nigoni* which was excluded due to the lack of a confident ortholog. Overall, NPR‐1 is relatively well conserved across the clade.

Given the variation in the social feeding behaviors in the *Caenorhabditis* genus, we considered what mechanisms might drive species differences. Morphological differences likely drive some of the changes; however, there has been very little characterization in most of the species we assayed. There are reports on the development of the vulva (Félix et al. 2000; Sharanya et al. 2012; Sommer and Sternberg 1994), tail morphology (Emmons 1992; Fitch 1997; Slos et al. 2017), and body size (Flemming et al. 2000) of some of these species. The lack of species morphological characterization is especially apparent when considering the nematode nervous systems, with the only other *Caenorhabditis* species with any significant neuronal characterization being *C. briggsae* and *C. tropicalis* (Ortiz et al. 2006; Toker et al. 2025). To bypass the lack of knowledge of neuronal circuits within the clade, we leveraged known genetic drivers of social feeding behavior in *C. elegans* , notably the neuropeptide receptor NPR‐1. To compare *npr‐1* across species, we first queried both existing and draft genome assemblies for orthologs using OrthoFinder (Emms and Kelly 2019). Given the size and relative domain conservation of the NPR family of genes in *C. elegans*, we were surprised to identify a single ortholog of NPR‐1 in all species except for *C*. *nigoni*, which had multiple related paralogues and was thus excluded from our analysis. To characterize sequence conservation among species, we used CLUSTAL V5.0 analysis to perform a multiple sequence alignment to produce an NPR‐1 alignment (Figure 5A). The alignment showed there is a high level of conservation among the species, even when considering the most evolutionary divergent species, like *C*. *monodelphis*. We queried three residues in NPR‐1 that when mutated have been shown to induce social behaviors (de Bono and Bargmann 1998) and found them to be well conserved and wild type.

## 4 Discussion

Here, we examined social foraging behaviors of an evolutionarily diverse clade of *Caenorhabditis* nematodes to better understand how such behaviors differ and may have evolved between species. Across these divergent species of nematodes, we noted several surprising trends within social feeding behaviors that underpin a shared feeding strategy. Notably, we confirmed *C. elegans* (*npr‐1*) is a relative extreme compared to several wild isolates and other species. In contrast, most of the species we assayed (including the wild *elegans* isolate CB4856 “Hawaiian” strain) showed intermediate levels of aggregation, while basal taxon *C*. *monodelphis* showed little to no aggregation, not dissimilar to *C. elegans* N2, which is known to be solitary due to a mutation in the *npr‐1* gene. Furthermore, we uncovered a surprising trend when examining bordering behavior among species, notably that the phenotype declines the further away the species is in relatedness to *C. elegans* . While this trend is not perfect, it highlights the idea that N2 “Bristol” *C. elegans* may represent a behavioral outlier when compared with wild *C. elegans* isolates and most other species.

In characterizing two social feeding behaviors, we identified unanticipated levels of variation both among and within species, suggesting the assays are subject to one or more additional variables that may have different contributions between species. The variation in several species suggests that an active mechanism drives heterogeneity in these behaviors and could be due to genetic heterogeneity between individuals that is maintained through selection pressure. *C. elegans* strains have been artificially selected and lab‐evolved (in part because they reproduce clonally), while most of the other species represent more heterogeneous genetic populations. The nature of *C. elegans* evolution in the lab raises the possibility that their sensitivity to environmental changes has been altered compared to more wild species, thus allowing them to react more predictably to change. Several species have been backcrossed and inbred extensively from their wild isolates, effectively rendering them lab strains (Table 1) after undergoing selection for domestication. Some of the species likely retain higher levels of genetic heterogeneity, especially among genetic elements such as single nucleotide variations (SNPs). While the vast majority of SNPs are unlikely to be the causative agent directly underlying a phenotypic change, single nucleotide changes can drastically alter behavior, as highlighted by single nucleotide variation in the neuropeptide receptor NPR‐1. Many wild isolates of *C. elegans* naturally aggregate and display social feeding, but in the lab N2 Bristol strain, a single adenine to guanine substitution in NPR‐1 is sufficient to produce a gain of function phenotype, eliminating social behavior and inducing solitary feeding (de Bono and Bargmann 1998). Lastly, genetic variants present within individuals may have a modulatory effect within particular behavioral circuits, especially if they are closely associated with critical neuronal genes or regulatory elements (Lee et al. 2024). This concept is highlighted in recent work showing that mutations in the *daf‐19* transcription factor dramatically alter chemosensory and oxygen‐sensing circuits and disrupt social feeding behaviors in both *C. elegans* and *P. pacificius* (Moreno et al. 2018).

Outside of genetic mechanisms, some of the variation observed in social feeding behaviors is likely a result of the lab environment in which these assays were performed. In the lab, we make every effort to maintain temperature at 20°C with low to moderate humidity, which is ideal for the growth and maintenance of lab strains like *C. elegans* and *C. briggsae* . However, many of the other *Caenorhabditis* species were isolated from different climates and environments and likely underwent adaptation of their environmental‐sensory circuits to these divergent conditions in contrast to those in which we assayed them. Therefore, we can speculate that context‐specific evolution may impact their adaptability to less favorable environments and increase variation among populations. While testing ecologically relevant temperatures and humidity levels for each species is logistically beyond the scope of this study, one might imagine that social feeding behaviors would be altered in some species if those conditions more accurately reflect those in the wild.

Furthermore, the food substrate used here is resoundingly artificial, using a monoculture of a lab strain of *E. coli* (OP50) on an NGM surface is hardly representative of the natural ecology of wild feeding substrates. When considering the natural environments in which these nematodes reside, the microbiomes can be dramatically heterogeneous depending on location. Most natural feeding substrates contain a range of bacterial species, some of which are viable food sources and some that may represent nematode pathogens. An additional layer of complexity arises when one considers differences in local microbiomes across nematode species and different ecological niches. To reconcile these differences, recent research has attempted to characterize food choice preferences in *C. elegans* . Early reports defined *C. elegans* ' preference for species such as *Bacillus* (Abada et al. 2009), while more recent work shows that *C. elegans* have little preference and foraging behaviors depend primarily on bacterial density (Madirolas et al. 2023). In addition, even within a single species such as *E. coli* , *C. elegans* tend to prefer nutrient‐dense strains, likely to supplement for essential nutrients, and potentially leading to changes in metabolism (Abada et al. 2009; Brooks et al. 2009). It is not surprising then that different food sources can lead to dramatic variations in behavior. For example, the attraction/avoidance response to food allows the animals to avoid potentially pathogenic bacteria while optimizing nutrient uptake (Meisel and Kim 2014; Lei et al. 2024). Lastly, *C. elegans* have been shown to modulate their feeding behaviors based on the density of food, where both solitary and social strains disperse when food density is strongly diminished (de Bono et al. 2002), likely as part of a codependent circuit detecting both starvation cues and increased oxygen (Rogers et al. 2006).

The last major factor potentially influencing these behaviors is chemical signaling in the form of environmental secretions and touch stimuli. Although the nature of these chemical modulators is widely unknown, they represent an intriguing mechanism by which individuals can modulate interactions with other animals and their environment. For example, several species of nematodes have complex and diverse secretomes, which they use to modulate interactions with their environment. Several studies have shown that parasitic nematodes secrete protein or chemical modulators to downregulate their host's immune response (Tritten et al. 2021). Similarly, the *C. elegans* genome encodes a large number of putative secreted proteins (Suh and Hutter 2012), some of which were isolated via mass spectrometry, identifying a number of novel and nematode‐specific protein factors of unknown function. In parallel, the chemical secretome of *C. elegans* revealed a complex biochemical landscape, heavily enriched in putative pheromones, especially ascarosides (Zhang et al. 2013). Given the unknown function of many of these proteins and chemicals it is likely that a subset influences the behaviors of both individuals and populations. Several ascaroside pheromones are well documented to alter population behaviors, modulating hermaphrodite‐male attractions and repulsion, mating, and dauer formation in the absence of food (Edison 2009). Furthermore, pheromone secretion detected in the environment can induce the production of EVs in neuronal tissues of the affected animals, potentially altering behavior (Szczepańska et al. 2024). We must therefore consider that unknown signaling molecules are modulating the interaction between individuals in our social feeding assays. Among the individuals of the same species, these signals may provide a real‐time readout of the local sensory environment helping animals to respond and alter their behavior accordingly. Among individuals of different species, the interactions are potentially even more complex. Differences in pheromone usage or chemical secretome might produce novel interactions and interesting behavioral variations.

Overall, this work represents the first broad characterization of social feeding behaviors across a large and evolutionarily ancient lineage of nematodes, and complements recent characterization of social feeding behaviors in wild *C. elegans* populations (Kang et al. 2025). Given the range of distinct environmental conditions in which these species naturally reside, it may not be surprising that we observed variation among species across several behavioral paradigms. The current circuit mechanism underlying the social feeding behaviors is still trying to fully reconcile how behavior is tuned in response to various sensory cues like food identity/density (de Bono et al. 2002; Abada et al. 2009; Brooks et al. 2009) and O_2_/CO_2_ balance (Gray et al. 2004). Complicating matters is recent evidence in *P. pacificus* showing that single cues such as elevation (and corresponding depletion of O_2_) can override the established behavioral paradigm and introduce variation (Moreno et al. 2016). Context‐dependent variation complicates dissection of the genetic and molecular mechanisms of behavioral variation, but presents an exciting opportunity to study real‐time plasticity of behaviors and neuronal circuits.

## Author Contributions

**Dustin Haskell:** conceptualization (equal), formal analysis (equal), investigation (lead), methodology (lead), supervision (equal), writing – original draft (equal). **Jhelaine Palo:** investigation (supporting), methodology (supporting). **Reina F. H. Eugene:** investigation (supporting), methodology (supporting). **Christopher R. L. Large:** data curation (supporting), investigation (supporting), methodology (supporting), writing – review and editing (supporting). **Michael P. Hart:** conceptualization (equal), investigation (supporting), methodology (supporting), project administration (lead), supervision (lead), visualization (equal), writing – original draft (equal), writing – review and editing (equal).

## Conflicts of Interest

The authors declare no conflicts of interest.

## Supporting information


**Figure S1:** Aggregation behavior is diverse across *Caenorhabditis* clade. (A) Representative images of additional species in the *Caenorhabditis* clade. (B) Multiple representative images of *Caenorhabditis nigoni* and *Caenorhabditis japonica* showing variation in aggregation and bordering behaviors.

**Figure S2:** Quantification of the number of aggregating animals for species in the *elegans* and *japonica Caenorhabditis* clades combined.

## Data Availability

The data that support the findings of this study are available on DRYAD at http://doi.org/10.5061/dryad.w3r22814k.
