# Supplementary figures and images for: Variation in Social Feeding Behaviors and Interactions Among Caenorhabditis Nematodes
Source: Ecol Evol. 2025 Nov 16;15(11):e72522. doi: 10.1002/ece3.72522 (PMC12620053; doi:10.1002/ece3.72522)

**A**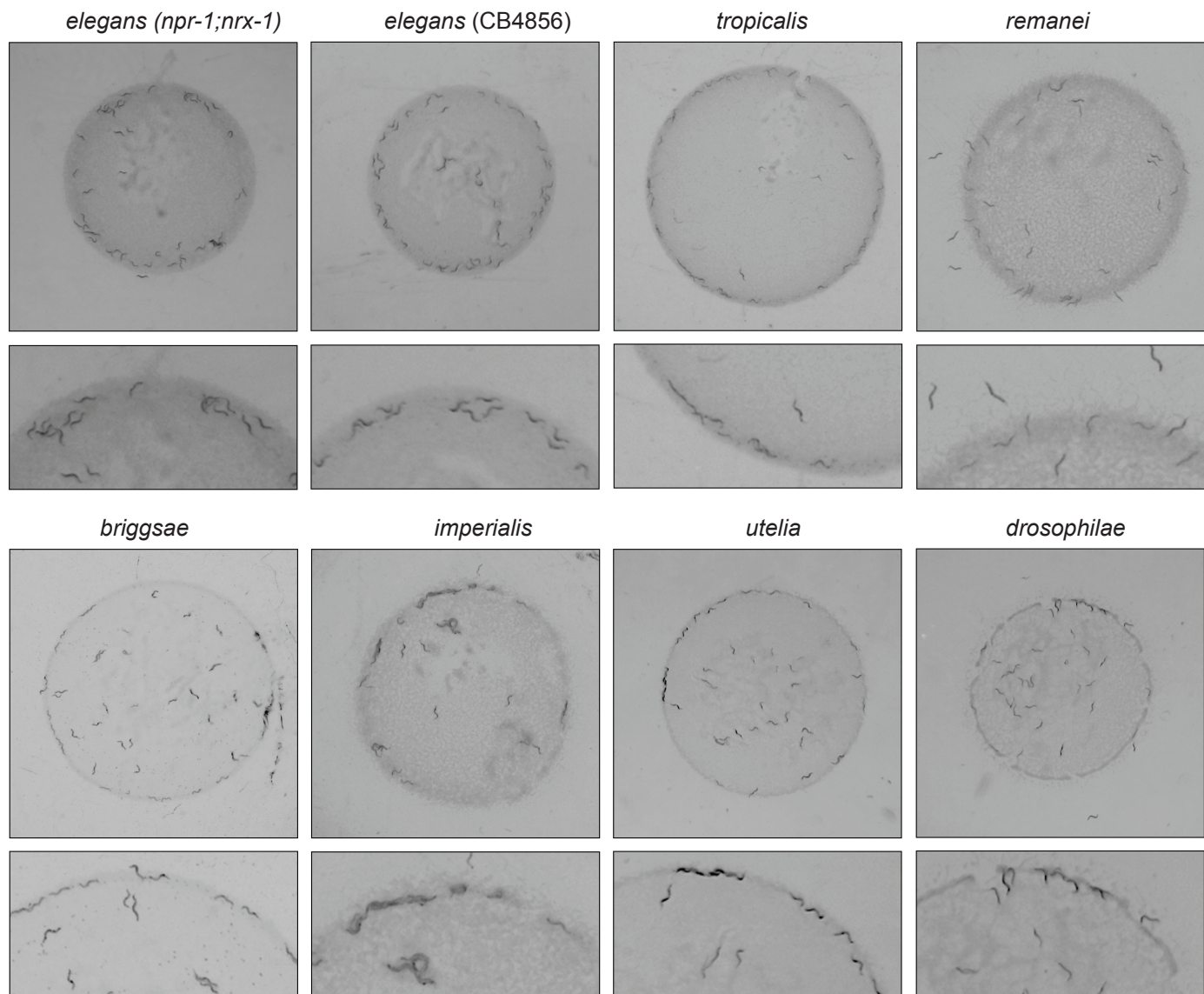**B**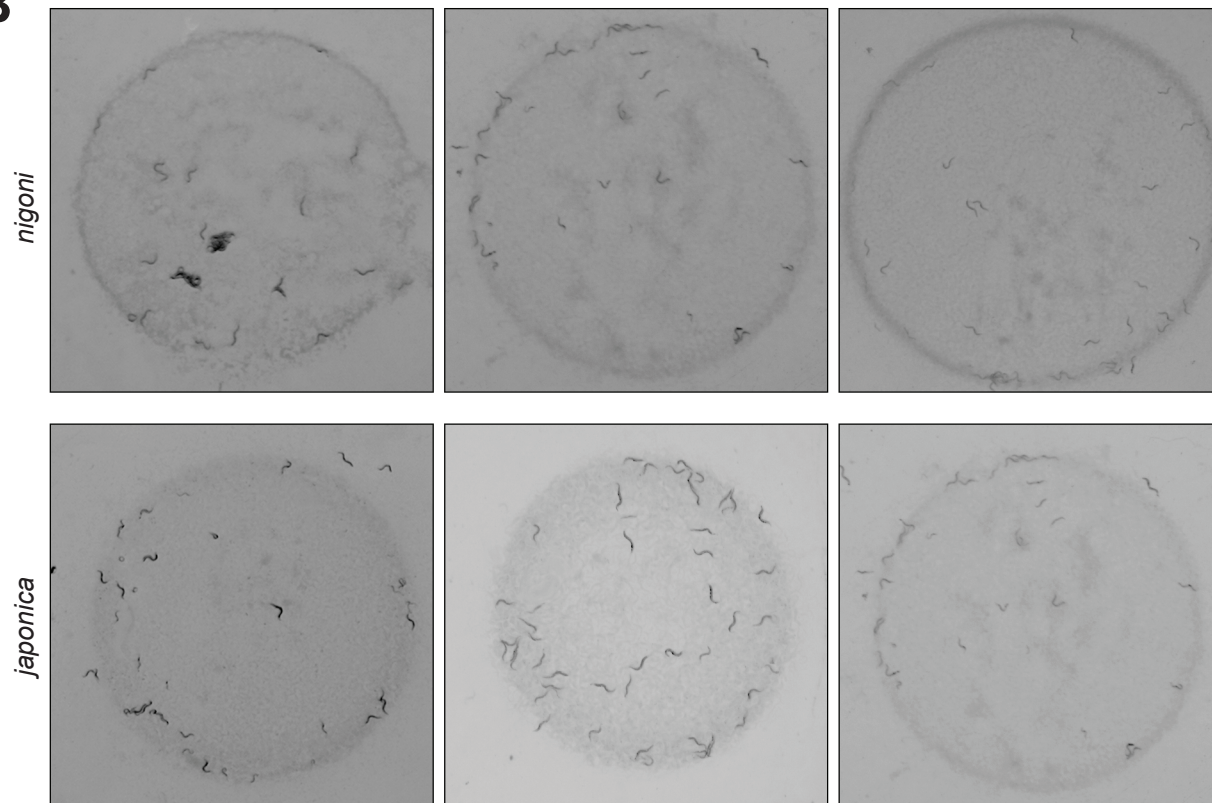

Supplement: Supplementary file 1 — Figure S1: Aggregation behavior is diverse across Caenorhabditis clade. (A) Representative images of additional species in the Caenorhabditis clade. (B) Multiple representative images of Caenorhabditis nigoni and Caenorhabditis japonica showing variation in aggregation and bordering behaviors. [file ECE3-15-e72522-s001.pdf]

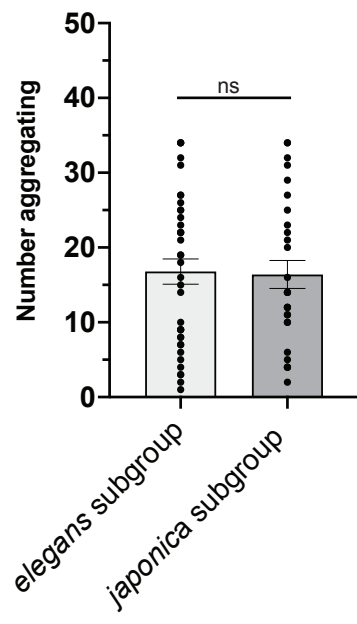

Supplement: Supplementary file 2 — Figure S2: Quantification of the number of aggregating animals for species in the elegans and japonica Caenorhabditis clades combined. [file ECE3-15-e72522-s002.pdf]
